# A randomized trial to assess the biological activity of short-term (pre-surgical) fulvestrant 500 mg plus anastrozole versus fulvestrant 500 mg alone or anastrozole alone on primary breast cancer

**DOI:** 10.1186/bcr3393

**Published:** 2013-03-05

**Authors:** John FR Robertson, J Michael Dixon, D Mark Sibbering, Ali Jahan, Ian O Ellis, Eddie Channon, Pauline Hyman-Taylor, Robert I Nicholson, Julia MW Gee

**Affiliations:** 1 Graduate Entry Medicine and Health School (GEMS), University of Nottingham, Royal Derby Hospital, Uttoxeter Road, Derby, DE22 3DT, UK; 2 Breast Unit, Western General Hospital, Crewe Road South, Edinburgh, EH4 2XU, UK; 3 Breast Unit, Royal Derby Hospital, Uttoxeter Road, Derby, DE22 3DT, UK; 4 Division of Surgery, Kingsmill Hospital, Mansfield Road, Sutton-in-Ashfield, NG17 4JL, UK; 5 School of Molecular Medicine, A Floor, West Block Queen's Medical Centre, University of Nottingham, Nottingham, NG7 2UH, UK; 6 Chirostat Statistical Consulting, University Boulevard, Beeston, Nottingham, NG9 2GJ, UK; 7 School of Pharmacy and Pharmaceutical Sciences, Redwood Building, King Edward VII Avenue, Cardiff University, Cardiff, CF10 3NB, UK

## Abstract

**Introduction:**

Fulvestrant shows dose-dependent biological activity. Greater estrogen-receptor (ER) blockade may feasibly be achieved by combining fulvestrant with anastrozole. This pre-surgical study compared fulvestrant plus anastrozole versus either agent alone in patients with ER-positive breast cancer.

**Methods:**

In this double-blind, multicenter trial, 121 patients received fulvestrant 500 mg on Day 1 plus anastrozole 1 mg/day for 14 to 21 days (F + A); fulvestrant plus anastrozole placebo (F); or fulvestrant placebo plus anastrozole (A), 2 to 3 weeks before surgery. ER, progesterone-receptor (PgR) and Ki67 expression were determined from tumor biopsies before treatment and at surgery.

**Results:**

A total of 103 paired samples were available (F, *n *= 35; F+A, *n *= 31; A, *n *= 37). All treatments significantly reduced mean ER expression from baseline (F: -41%, *P *= 0.0001; F + A: -39%, *P *= 0.0001; A: -13%, *P *= 0.0034). F and F + A led to greater reductions in ER versus A (both *P *= 0.0001); F + A did not lead to additional reductions versus F. PgR and Ki67 expression were significantly reduced with all treatments (means were -34% to -45%, and -75% to -85%, respectively; all *P *= 0.0001), with no differences between groups.

**Conclusions:**

In this short-term study, all treatments reduced ER expression, although F and F + A showed greater reductions than A. No significant differences were detected between the treatment groups in terms of PgR and Ki67 expression. No additional reduction in tumor biomarkers with combination treatment was observed, suggesting that F + A is unlikely to have further clinical benefit over F alone.

**Trial registration:**

Clinicaltrials.gov NCT00259090.

## Introduction

Fulvestrant is an estrogen-receptor (ER) antagonist with no known agonist effects. It binds, blocks and accelerates degradation of the ER, leading to dose-dependent reductions in cellular ER and proliferation-related antigen Ki67 [1,2]. Unlike tamoxifen, which increases progesterone-receptor (PgR) expression, fulvestrant also induces dose-dependent reductions in PgR expression [1]. Fulvestrant-mediated reductions in tumor biomarkers are indicative of anti-estrogenic and anti-proliferative effects [1,3,4], and may be useful as surrogate endpoints of clinical efficacy. The registration trials of fulvestrant (0020 and 0021) provided the first indication of its dose-related anti-tumor activity. Fulvestrant 250 mg was not significantly different from the aromatase inhibitor (AI) anastrozole, 1 mg daily, for the primary endpoint of time to progression in the treatment of postmenopausal women with advanced breast cancer who had recurred or progressed following prior endocrine therapy [5-7]. Although these trials initially incorporated a fulvestrant 125 mg treatment arm, an interim analysis showed insufficient evidence of clinical activity (no objective tumor responses and numerically lower time to progression (TTP)), and this arm was discontinued [5-8].

As ER down-regulation appears to be dose-dependent, it was hypothesized that ER antagonism may be enhanced by raising fulvestrant steady-state plasma concentrations to a higher level than those achieved by the 250 mg/month regimen [1,2]. In addition to this, the biological activity of fulvestrant 500 mg (fulvestrant 500 mg/month plus 500 mg on Day 14 of Month 1) versus 250 mg (fulvestrant 250 mg/month) was investigated in the Neoadjuvant Endocrine therapy for Women with Estrogen-Sensitive Tumors (NEWEST) study. The higher fulvestrant dose was associated with significantly greater down-regulation of Ki67 at Week 4 (primary endpoint), as well as similar reductions in ER and PgR protein expression. The higher fulvestrant dose also produced numerically higher tumor response rates [9].

The clinical activity of fulvestrant 500 versus 250 mg was further compared in the phase III COmparisoN of Faslodex In Recurrent or Metastatic breast cancer (CONFIRM) study, in which a significantly longer TTP was reported with the 500 mg dose (hazard ratio (HR) = 0.80; *P *= 0.006) [10].

Other questions posed were, first, whether a 500 mg regimen might be better than an AI, given that fulvestrant 250 mg had been equivalent to anastrozole; and second, whether there was any value in combining an AI and fulvestrant, either at the 250 or 500 mg dose. The rationale for the latter approach was that combining fulvestrant with an estrogen-lowering agent may lead to enhanced ER blockade and anti-tumor activity. This possibility was examined in the Fulvestrant loading dose and Anastrozole in Combination Trial (FACT), in which patients at first relapse received either a loading-dose regimen of fulvestrant (500 mg on Day 0; 250 mg on Days 14 and 28; then 250 mg monthly thereafter) plus anastrozole (1 mg daily), or anastrozole alone [11]. There were no significant differences between the two groups in the primary endpoint of TTP (HR = 0.99; *P *= 0.91) or in any of the secondary endpoints [11]. Critically, there was no underlying biomarker study, and FACT did not compare the combination of fulvestrant plus anastrozole versus fulvestrant alone. In the recently reported SWOG S0226 study, Mehta and colleagues report that the combination of the fulvestrant 250 mg loading-dose regimen with anastrozole was associated with improved progression-free survival versus anastrozole alone in postmenopausal women with untreated advanced breast cancer, indicating that the combination treatment may be effective in some patients [12].

This pre-surgical study was thus designed to compare the biological activity and safety of fulvestrant 500 mg versus anastrozole versus the combination (fulvestrant 500 mg plus anastrozole) as pre-surgical treatment in women with ER-positive breast cancer. This study was conducted in parallel with the Fulvestrant fIRst-line Study comparing endocrine Treatments (FIRST) phase II trial, which compared the clinical efficacy of fulvestrant 500 mg versus anastrozole for the first-line treatment of advanced hormone receptor-positive breast cancer [13]. The primary endpoint of FIRST was clinical benefit rate (CBR), which was numerically but not statistically greater for fulvestrant 500 mg compared with anastrozole (72.5% versus 67.0%), with the odds ratio (OR = 1.30; *P *= 0.386) in favor of fulvestrant. The secondary endpoint (TTP) was, however, significantly longer for fulvestrant 500 mg (median TTP not reached for fulvestrant *v *12.5 months for anastrozole; HR = 0.63; *P *= 0.0496) [13]. The superior clinical efficacy seen in FIRST makes the present biomarker study even more important, both for its potential to provide a biological rationale for why fulvestrant 500 mg might be better than anastrozole and also to provide an insight into whether adding anastrozole to fulvestrant 500 mg might be a treatment strategy worth pursuing.

## Materials and methods

### Study design and patients

**Figure 1:**
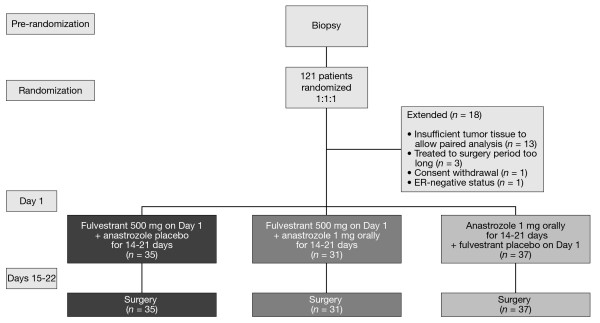
**Study design**. ER, estrogen receptor.

Trial 0057 was a phase II, double-blind, randomized, multicenter trial (9238IL/0057; NCT00259090). Patients were postmenopausal women (>12 months since the last menstrual period and/or castrate levels of follicle-stimulating hormone (>40 IU/liter)) with histologically or cytologically confirmed ER-positive, primary breast cancer (T_1_, T_2 _or T_3_). Patients had to be fit for surgery within one month and have a tumor large enough to provide sufficient biopsy samples. Patients were excluded from the trial if they had evidence of metastatic disease; received prior endocrine treatment for breast cancer, prior neoadjuvant chemotherapy or prior radiotherapy to the primary tumor; abnormal laboratory values; any severe concurrent condition; received hormone replacement therapy within the past four weeks; a history of disease affecting steroid metabolism; a history of bleeding diathesis, thrombocytopenia or a need for long-term anti-coagulant therapy; risk of human immunodeficiency virus, hepatitis B or hepatitis C transmission; evidence of severe systemic disease; baseline hematology or clinical chemistry outside the normal range; or any other reason that could jeopardize the protocol. Prior to surgery of curative intent, patients were randomized 1:1:1 to receive a single administration of fulvestrant 500 mg (2 × 250 mg on Day 1 via intramuscular injection) plus anastrozole (1 mg orally once daily for 14 to 21 days), or fulvestrant 500 mg plus anastrozole placebo or anastrozole plus fulvestrant placebo (Figure 1).

All patients provided written informed consent. The study was performed in accordance with the Declaration of Helsinki and was consistent with International Conference on Harmonization of Technical Requirements for Registration of Pharmaceuticals for Human Use (ICH) Good Clinical Practice. The study protocol, patient consent forms and information sheets were approved by the relevant independent ethics committee (Nottingham Ethics Committee 1; ethics reference number EC02/170) and institutional review boards.

### Study assessments

The primary endpoints were treatment effects on ER, PgR and Ki67 expression in tumor biopsy samples obtained pre-treatment and during surgery. These three tumor biomarkers were considered equally important indicators of fulvestrant activity. Their expression was determined by immunohistochemistry on sections of formalin-fixed, paraffin-embedded tissue, assaying matched sequential biopsy samples together for each patient. Antigen retrieval for ER and PgR was performed by pressure cooking in 0.01 M sodium citrate buffer, pH 6.0. Optimal ER staining was achieved with primary antibody clone 6F11 (NCL-ER-6F11, Novocastra, Newcastle upon Tyne, UK). For maximal PgR staining sensitivity, sections were co-incubated with primary antibody clones 636 (M3569, Dako Ltd., Cambridge, UK) and 16 (VP-P976, Vector Laboratories, Burlingame, CA, USA). Clone MIB-1 anti-Ki67 antibody (M7240, Dako Ltd.) was employed following microwave antigen retrieval in 0.01 M citric acid. The EnVision secondary antibody detection kit (Dako Ltd.) was used in all instances with DAB chromogen (Dako Ltd.). All biomarker measurements were performed at the Tenovus Centre for Cancer Research by two research scientists (JMWG and PF) experienced in H-score measurement. At least five fields were viewed simultaneously using a light microscope at x20 magnification and a representative field was then assessed by both researchers at x40 magnification using a dual viewing attachment. All samples consisted of at least 100 tumor epithelial cells. Reference to equivocal samples from a pathologist was available when required. As described previously [1], the percentage of ER- or PgR-positive tumor epithelial cell nuclei in each staining category (that is, negative -/-; very weak +/-; weak +; moderate ++; strong +++) were recorded for each sample by consensus of the two assessors. Results were expressed as the H-score, where H-score = ((0.5 × % +/-) + (1 × +) + (2 × % ++) + (3 × % +++)), with a range of 0 to 300. Ki67 index was also assessed by light microscopy and consensus results for each sample were expressed as the percentage of positively stained tumor epithelial cells, as described previously [1]. To monitor assay performance, breast-cancer samples of known ER, PgR or Ki67 content were included as positive controls in every assay.

Rates of breast-conserving surgery, clinical response rate and pathological response rates were not collected prospectively, as these data were not expected to provide meaningful results in this short-term study.

Tolerability was a secondary endpoint and was assessed by recording the incidence of adverse events (AEs) following treatment and surgery. AEs were classified according to the Medical Dictionary for Regulatory Activities.

### Statistical analysis

Based on data from our previous fulvestrant study [1], sample size calculations indicated that 30 patients per arm were needed to detect relevant differences between treatment groups with 80% power (38.1 for ER; 21.5 for PgR; and 64.4 for Ki67), using a two-sided significance level of 5%. This is consistent with other similar studies, in which patient numbers are usually small. For the analysis of Ki67, the detectable ratio was 0.644 and so the study was powered to detect a difference between changes of, for example, -75% and -91%. The study was not powered to detect smaller changes between treatment groups, and sample sizing was a balance between the ability to detect differences between arms and the challenge of patient recruitment across the four centers. Analysis of covariance (ANCOVA), with terms in the model for treatment and center, and baseline values as a covariate, was performed to compare the mean H-score post-treatment. If the test for overall treatment effect was significant at the 5% level, then all pairwise comparisons were made (this is Fisher's protected Least Significant Difference test). The overall treatment effect and pairwise comparison calculations were also performed using a Bonferroni correction, to allow for having three primary endpoints. For Ki67, the data showed evidence of non-normality, so all values were log-transformed (after adding a constant of 1) and the results back-transformed to the original scale. ANCOVA was also used to assess differences between mean H-score at baseline and post-treatment within each treatment group using individual change values. As an aid to interpretation, mean changes from baseline have been converted to percentage of initial staining (by dividing mean changes by the overall mean at baseline).

The number of tumors that showed a reduction in ER and PgR was also deemed to be of interest. In a descriptive analysis, the number of individual patients with reductions from baseline in ER or PgR H-score was tabulated.

## Results

### Patients

In total, 121 patients were randomized at four centers in the UK and 18 patients were omitted from the final analysis (Figure 1). The first patient was randomized on 5 January 2006 and the final patient completed the trial on 9 October 2008. Paired samples from 103 patients were available for analysis: 35 patients treated with fulvestrant 500 mg alone, 31 patients treated with fulvestrant 500 mg plus anastrozole, and 37 patients treated with anastrozole alone. Mean age was 65.7 years (range 50 to 88 years) and the majority of patients had grade-2 tumors (Table 1).

**Table 1:** Patient demographics

	Fulvestrant 500 mg	Fulvestrant 500 mg + anastrozole 1 mg	Anastrozole 1 mg
*n*	35	31	37
Age, years			
Mean	67.9	64.2	64.8
Standard deviation	8.5	10.2	8.7
Tumor grade^a^			
1	8	9	8
2	23	16	25
3	4	4	4

^a^Two patients in the fulvestrant 500 mg plus anastrozole group had bilateral breast tumors of differing grades and are not included in the above baseline grade table.

### Tumor biomarker expression

ER H-scores were significantly reduced from baseline by -41% in the fulvestrant 500 mg group (*P *= 0.0001), -39% in the fulvestrant 500 mg plus anastrozole group (*P *= 0.0001) and -13% in the anastrozole group (*P *= 0.0034) (Table 2).

**Table 2:** Summary of ER, PgR and Ki67 labeling index data

	Fulvestrant 500 mg	Fulvestrant 500 mg + anastrozole 1 mg	Anastrozole 1 mg
**ER**			
*n*	35	31	37
Pre-treatment mean H-score	187.7	184.2	192.2
Post-treatment mean H-score	111.9	115.8	164.2
Change (post-treatment), % (SEM)	-41 (4)	-39 (5)	-13 (4)
Comparison versus baseline	*P *= 0.0001	*P *= 0.0001	*P *= 0.0034
**PgR**			
*n*	33	28	33
Pre-treatment mean H-score	145.7	141.7	157.6
Post-treatment mean H-score	97.9	81.1	93.8
Change (post-treatment), % (SEM)	-34 (6)	-45 (7)	-37 (6)
Comparison versus baseline	*P *= 0.0001	*P *= 0.0001	*P *= 0.0001
**Ki67**			
*n*	35	31	37
Pre-treatment mean index	17.1	17.8	16.2
Post-treatment mean index	4.2	3.3	2.6
Change (post-treatment), % (SEM)	-75 (9)	-81 (7)	-85 (5)
Comparison versus baseline	*P *= 0.0001	*P *= 0.0001	*P *= 0.0001

ER, estrogen receptor; PgR, progesterone receptor; SEM, standard error of the mean.

**Figure 2:**
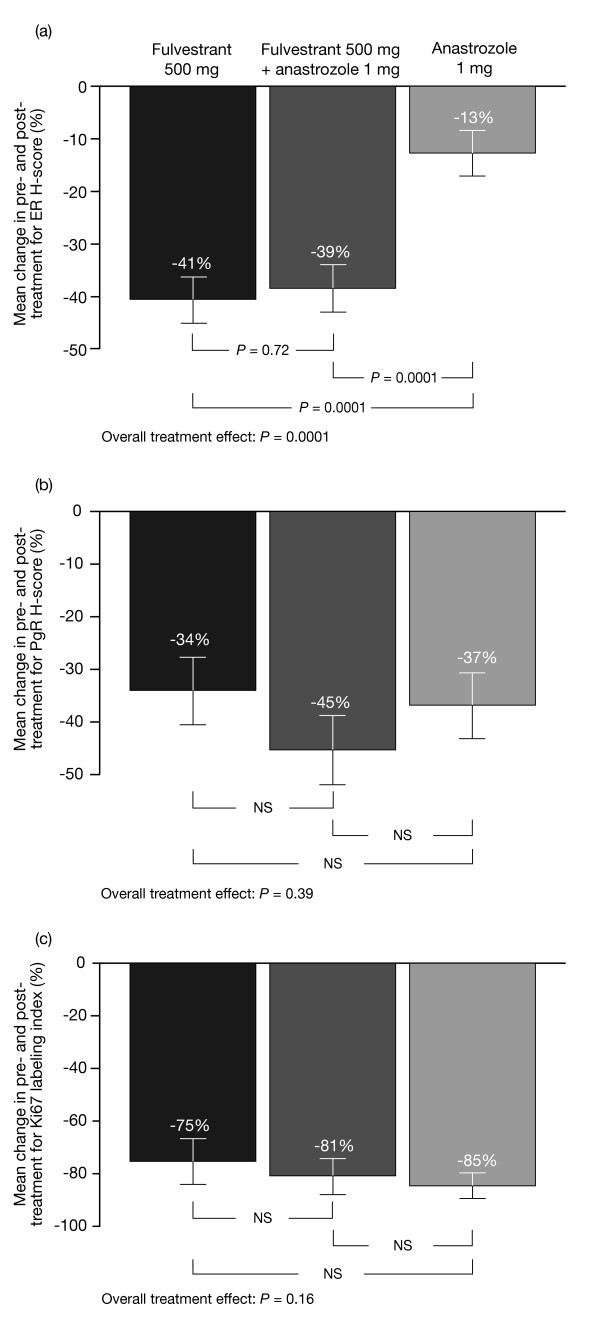
**Mean change in pre- and post-treatment data**. **(a) **ER H-score, **(b) **PgR H-score and **(c) **Ki67 labeling index. Where the overall tests for treatment effect were not significant for total PgR H-score and Ki67 index, pairwise comparisons were deemed non-significant. Error bars represent standard error of the mean. ER, estrogen receptor; NS, not significant; PgR, progesterone receptor.

**Figure 3:**
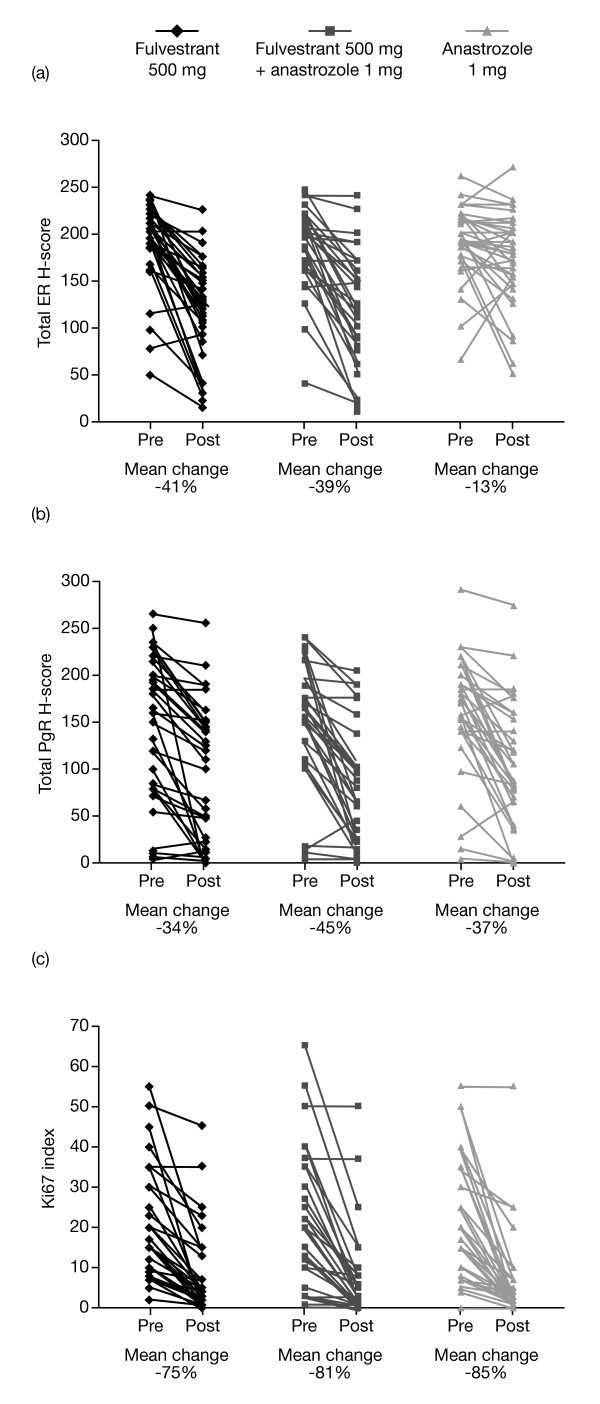
**Scatter plots of individual pre- versus post-treatment data**. **(a) **ER, **(b) **PgR and **(c) **Ki67. ER, estrogen receptor; PgR, progesterone receptor.

Fulvestrant 500 mg alone and the combination of fulvestrant 500 mg plus anastrozole led to greater reductions in ER H-score compared with anastrozole alone (both *P *= 0.0001) (Figures 2a and 3a). There was no significant difference in ER H-score reductions between fulvestrant 500 mg plus anastrozole versus fulvestrant 500 mg alone (*P *= 0.72).

Nine patients with a PgR H-score of 0 at baseline were omitted from the analysis. PgR H-scores were significantly reduced from baseline by all treatments: -34% with fulvestrant 500 mg, -45% with fulvestrant 500 mg plus anastrozole and -37% with anastrozole alone (all *P *= 0.0001) (Table 2). There were no significant between-treatment differences in reduction of PgR (Figures 2b and 3b). Similarly, Ki67 expression (assessed for all patients) was significantly and substantially reduced from baseline in all groups (-75%, -81% and -85% for fulvestrant 500 mg alone, fulvestrant 500 mg plus anastrozole, and anastrozole alone, respectively; all *P *= 0.0001) (Table 2), with no significant differences between treatments (Figures 2c and 3c). The residual level of proliferation after any of these treatments was invariably low, reflected in the post-treatment mean index levels (Table 2).

The significance levels for reduction in ER and PgR H-score and Ki67 expression remained unchanged when the Bonferroni correction was applied to allow for having three primary endpoints (that is, using a significance level of *P *<0.017).

When changes in ER H-score were assessed, 86% of patients in the fulvestrant 500 mg group and 77% of patients in the fulvestrant 500 mg plus anastrozole group experienced a decrease in H-score of >20 from baseline. Fewer patients in the anastrozole group (35%) experienced a decrease in H-score of >20 from baseline. The percentage of patients exhibiting reductions in PgR H-scores was similar across the three groups; 64%, 75% and 73% of patients in the fulvestrant 500 mg, fulvestrant 500 mg plus anastrozole, and anastrozole treatment groups, respectively, experienced a reduction in PgR H-score of >20 from baseline.

### Tolerability

The incidence of AEs was similar in all treatment groups, with 68% of the patients experiencing at least one AE with fulvestrant 500 mg, 68% with fulvestrant 500 mg plus anastrozole and 73% with anastrozole alone. The most common AE in patients who received fulvestrant 500 mg alone or anastrozole alone was hot flash (18% and 20%, respectively) (Table 3). In the fulvestrant 500 mg plus anastrozole group, the most frequent AEs were headache (10%) and pain in an extremity (10%).

**Table 3:** Most common AEs occurring during the study

	Patients experiencing an AE,^a ^*n *(%)		
	**Fulvestrant** **500 mg**	**Fulvestrant** **500 mg + anastrozole 1 mg**	**Anastrozole 1 mg**

**Hot flash**	7 (18)	3 (8)	8 (20)
**Headache**	3 (8)	4 (10)	3 (8)
**Pain in extremity**	0 (0)	4 (10)	2 (5)
**Constipation**	3 (8)	1 (3)	2 (5)
**Injection-site pain**	2 (5)	1 (3)	3 (8)

**Any AE**	27 (68)	27 (68)	29 (73)

^a^Data for the intent-to-treat population. One patient who received no treatment was excluded. AE, adverse event.

There were no serious AEs (SAEs) in the fulvestrant group, three SAEs in the fulvestrant 500 mg plus anastrozole group (atrial fibrillation, hospitalization for right mastectomy and procedural complication due to skin flap failure), and two SAEs in the anastrozole group (subcutaneous abscess and pancytopenia). No SAEs were considered by the investigator to be treatment-related. Two patients in the anastrozole group withdrew from the study due to AEs: pancytopenia (SAE) and cardiac arrhythmia (non-SAE).

## Discussion

Previous neoadjuvant studies have compared selective ER modulators, such as tamoxifen, with AIs, such as anastrozole [14] and letrozole [15]. To our knowledge, this study is the first to compare directly, in a randomized trial, the biological activity of a selective ER antagonist, such as fulvestrant, versus an AI in a pre-surgical setting. Furthermore, the dose of fulvestrant used was 500 mg, which is now the recommended dose in many countries. This study is also the first to compare the activity of fulvestrant 500 mg with and without anastrozole. Following treatment, ER H-scores were significantly reduced from baseline in all groups; however, there were greater reductions with fulvestrant 500 mg and fulvestrant 500 mg plus anastrozole, compared with anastrozole alone. These findings are entirely in accordance with the known mechanism of action (MoA) of these two agents: fulvestrant as a selective ER antagonist reduces tumor ER protein levels, while anastrozole reduces estradiol levels and, therefore, signaling through the ER, but has little or no inhibitory effect on ER levels. No additional reduction in ER expression was observed when fulvestrant was combined with anastrozole, compared with fulvestrant alone.

PgR expression levels were also significantly reduced from baseline in all groups, but there were no significant differences between fulvestrant plus anastrozole and either agent alone. Again, this is in agreement with what is known about the MoA of these drugs. PgR is an estrogen-inducible protein, and removal of ER signaling either by ER down-regulation or by blockade of estradiol synthesis leads to reduced PgR levels. Similarly, either MoA can decrease tumor cell proliferation. Although all treatments impacted substantially on ER function and proliferation, there was still some ER and PgR expression remaining in the post-fulvestrant treatment biopsies.

The fact that combining two endocrine agents did not result in increased reduction of PgR or Ki67 levels confirms that they act with equivalent magnitude on the same signaling pathway, but at different points. The reductions in tumor biomarkers observed after anastrozole treatment are also consistent with a recent randomized phase II study which reports that anastrozole and letrozole led to equally significant reductions in Ki67 expression [16]. Ellis and colleagues also reported that neoadjuvant treatment with an AI was effective at improving clinical response rates and surgical outcomes in postmenopausal breast cancer.

One of the strengths of this randomized pre-surgical study is that double-blind procedures were extended until all biomarker measurements had been made before the treatment codes were broken.

The initial rationales for this study were that fulvestrant 500 mg might be biologically more potent than an AI and also that a synergistic effect of combining the two could be achieved. As fulvestrant competes with estradiol for ER binding, reducing plasma estrogen levels using anastrozole could feasibly increase fulvestrant-ER binding and increase its efficacy. Using an intratumoral, aromatase-transfected xenograft model, fulvestrant plus anastrozole was found to delay tumor growth more effectively than either agent alone [17]. In addition, further reduction in ER levels and down-regulation of signaling proteins involved in the development of hormonal resistance (for example, insulin-like growth factor receptor 1 (IGF-1R), MAPK and AKT) were observed with the combination treatment. Despite these preclinical findings, our study found no biomarker evidence that the combined treatment had enhanced biological activity in patients with breast cancer. These results are in line with the FACT study, which reported no benefit in clinical endpoints with the combination of a loading-dose regimen of fulvestrant (250 mg) plus anastrozole versus anastrozole alone [11]. The recently described SoFEA trial also failed to show any efficacy benefit for the combination of fulvestrant with an AI. Equivalent PFS was demonstrated for fulvestrant (250 mg loading-dose regimen) plus anastrozole compared with fulvestrant alone in postmenopausal patients with advanced breast cancer following progression on non-steroidal AIs [18]. However, data from the SWOG S0226 study suggest that combination therapy as first-line treatment for advanced breast cancer was associated with efficacy benefits in some patients and may warrant further study [12]. For the primary endpoint, median PFS was 15.0 months for the combination therapy (fulvestrant 250 mg loading-dose regimen plus anastrozole) compared with 13.5 months for anastrozole alone (HR = 0.80; *P *= 0.007). The reason for the different outcomes in FACT and SWOG S0226 has not been fully established. However, this may in part be explained by the proportion of patients who had received prior adjuvant endocrine therapy, which was somewhat higher in the FACT trial. In a retrospective analysis of those patients who had received previous adjuvant tamoxifen treatment in the SWOG S0226 study (280/694; 40.3%), median PFS was 13.5 months in the combination group compared with 14.1 months in the anastrozole-alone group (HR = 0.89; *P *= 0.37). In those patients naive to prior tamoxifen therapy (414/694; 59.7%), median PFS was 17.0 months for the combination compared with 12.6 months for anastrozole alone (HR = 0.74; *P *= 0.006) [12]. Importantly, there was no fulvestrant-alone arm in SWOG S0226 and so it is not possible from this study to establish if the difference between the two arms is due to the fulvestrant 250 mg loading-dose regimen being better than anastrozole in the first-line setting or due to the combination of fulvestrant 250 mg and anastrozole.

Recently, a growing body of evidence has suggested that fulvestrant 500 mg would offer efficacy benefits over the existing 250 mg regimen. NEWEST was the first study to demonstrate a higher biological activity (depletion of ER, PgR and Ki67) for fulvestrant 500 mg versus 250 mg [9]. In addition, results from the CONFIRM study indicate greater clinical efficacy with the 500 mg regimen, without increased toxicity [10]. It, therefore, appears that fulvestrant 500 mg results in both increased biological activity and clinical efficacy.

The median day of biopsy across all patients in the present study was Day 18 following a single fulvestrant 500 mg dose. However, previously published data suggest that steady-state plasma fulvestrant levels are achieved after approximately 28 days with the fulvestrant 500 mg dose regimen (which also includes a 500 mg dose on Day 14) [8], and hence exposures following a single 500 mg dose would be lower than those achieved at steady state. This would suggest that the biological effect seen with a single fulvestrant 500 mg dose may be an underestimate compared with the fulvestrant 500 mg dose regimen approved for clinical practice.

The biological results from the present study may shed light on the results from FIRST, in which the secondary endpoint of TTP was significantly longer for fulvestrant 500 mg over anastrozole (*P *<0.05) [13], an effect that was maintained in prolonged follow-up data (HR = 0.66; *P *= 0.01) [19]. The primary endpoint showed a numerical, but not statistically significant, difference in CBR. In the current study, there was a greater decrease in ER (but not in Ki67) for fulvestrant 500 mg compared with anastrozole. The similar substantial initial decrease in proliferation (that is, Ki67) would be in keeping with the initial CBRs (*de novo *response) seen in FIRST. The improvement in TTP in FIRST occurred after six months (due to prolongation of acquired resistance in tumors in which initial clinical benefit was shown). In model systems, a mechanism implicated in acquired resistance to endocrine therapy (including estrogen deprivation) is cross-talk between ER and other growth factor pathways (for example, HER2, IGF-1R and downstream signaling kinases) [20-23]. The activity of residual ER (or growth factor signaling elements) following fulvestrant treatment remain largely unexplored, but the greater reduction in ER seen here with fulvestrant might feasibly hinder instigation of such cross-talk mechanisms and thus delay emergence of acquired resistance, compared with tumors treated with anastrozole alone.

When tolerability was assessed, the AE profile was similar in all treatment groups, with no emerging safety concerns for fulvestrant 500 mg. However, only very limited safety data were available due to the short treatment duration in the study. The safety profile of fulvestrant 500 mg has previously been described in FIRST and CONFIRM, where the safety profile of the higher dose was similar to that of the 250 mg dose. The comparable AE profile between fulvestrant 500 mg plus anastrozole and anastrozole alone was in keeping with the side-effect profiles of fulvestrant 250 mg plus anastrozole versus anastrozole alone reported in FACT [11]. There were, therefore, no new safety concerns for the higher-dose fulvestrant regimen, when used alone or in combination with anastrozole [10,13,24].

## Conclusions

This is the first direct comparison of a selective ER antagonist versus an AI, and reported greater down-regulation of ER with fulvestrant 500 mg compared with anastrozole. Reductions in Ki67 labeling index and PgR expression were comparable between the treatment groups. This study is also the only one thus far to compare fulvestrant 500 mg plus anastrozole versus fulvestrant 500 mg alone. It demonstrated no additional reductions in ER, PgR and Ki67 with the combination, adding to data indicating that combining other types of anti-estrogen (for example, tamoxifen) with an AI does not appear to provide additional clinical benefit over an anti-estrogen alone [25-27].

## Abbreviations

AE: adverse event; AI: aromatase inhibitor; ANCOVA: analysis of covariance; CBR: clinical benefit rate; CONFIRM: COmparisoN of Faslodex In Recurrent or Metastatic breast cancer; ER: estrogen receptor; FACT: Fulvestrant loading dose and Anastrozole in Combination Trial; HR: hazard ratio; IGF-1R: insulin-like growth factor receptor 1; MoA: mechanism of action; NEWEST: Neoadjuvant Endocrine therapy for Women with Estrogen-Sensitive Tumors; PgR: progesterone receptor; SAE: serious adverse event; TTP: time to progression

## Competing interests

JFRR has acted as a consultant on advisory boards for AstraZeneca and Bayer HealthCare, and received research funding from Amgen, AstraZeneca, Bayer HealthCare and Novartis. He has received honoraria for speaking at symposia organized by AstraZeneca and GlaxoSmithKline. He has provided expert testimony on fulvestrant before the European Medicines Agency. IOE is the Medical Director of Source Bioscience plc and has acted as a consultant on advisory boards for Roche. EC has received payment from Nottingham University using AstraZeneca research funding. RIN and JMWG currently hold research grants from AstraZeneca. JMD, DMS, AJ and PH-T have no conflicts of interest to declare. This was an investigator-initiated study, sponsored by the University of Nottingham and supported by a research grant from AstraZeneca Pharmaceuticals. The article-processing charge is being financed by AstraZeneca.

## Authors' contributions

JFRR and IOE designed the concept of this study. JFRR, JMD, DMS, AJ, IOE, EC, PH-T and JMWG collected and assembled the trial data. All biomarker immunohistochemistry was performed under the laboratory direction of JMWG. JFRR, EC, RIN and JMWG performed the data analysis and interpretation. All authors were involved in the writing of the manuscript and approved the final version of the manuscript.
